# Multidisciplinary Management of Pituitary Apoplexy

**DOI:** 10.1155/2016/7951536

**Published:** 2016-12-15

**Authors:** Adriana Albani, Francesco Ferraù, Filippo Flavio Angileri, Felice Esposito, Francesca Granata, Felicia Ferreri, Salvatore Cannavò

**Affiliations:** ^1^Unit of Endocrinology, Department of Clinical and Experimental Medicine, University of Messina, Messina, Italy; ^2^Unit of Neurosurgery, Department of Biomedical and Dental Sciences and Morphofunctional Imaging, University of Messina, Messina, Italy; ^3^Unit of Neuroradiology, Department of Biomedical and Dental Sciences and Morphofunctional Imaging, University of Messina, Messina, Italy; ^4^Unit of Ophthalmology, Department of Biomedical and Dental Sciences and Morphofunctional Imaging, University of Messina, Messina, Italy

## Abstract

Pituitary apoplexy is a rare clinical syndrome due to ischemic or haemorrhagic necrosis of the pituitary gland which complicates 2–12% of pituitary tumours, especially nonfunctioning adenomas. In many cases, it results in severe neurological, ophthalmological, and endocrinological consequences and may require prompt surgical decompression. Pituitary apoplexy represents a rare medical emergency that necessitates a multidisciplinary approach. Modalities of treatment and times of intervention are still largely debated. Therefore, the management of patients with pituitary apoplexy is often empirically individualized and clinical outcome is inevitably related to the multidisciplinary team's skills and experience. This review aims to highlight the importance of a multidisciplinary approach in the management of pituitary apoplexy and to discuss modalities of presentation, treatment, and times of intervention.

## 1. Introduction

Pituitary tumour-associated haemorrhage was described for the first time by Bailey in 1898, but only in 1950 Brougham et al. introduced the term pituitary apoplexy describing a case series of five patients [1]. According to recent epidemiological studies, pituitary apoplexy has prevalence of about 6.2 cases per 100000 inhabitants [2] and its incidence is estimated as 0.17 episodes per 100000 per year [3].

In more than 80% of the patients, pituitary apoplexy is often the first presentation of an underlying pituitary tumour (especially nonfunctioning adenomas) [4, 5]. It is estimated that the incidence of intralesional bleeding in a pituitary adenoma is five times higher than that in other intracranial neoplasms [6]. However, apoplexy can also occur in nonadenomatous lesions, such as hypophysitis [7, 8], craniopharyngioma, Rathke's cleft cyst, sellar tuberculoma [9], and sellar metastasis [10], or even in normal pituitary gland, as a consequence of sever hypovolemic events during the delivery or the puerperium (Sheehan's Syndrome) [11, 12].

## 2. Pathophysiology

**Figure 1:**
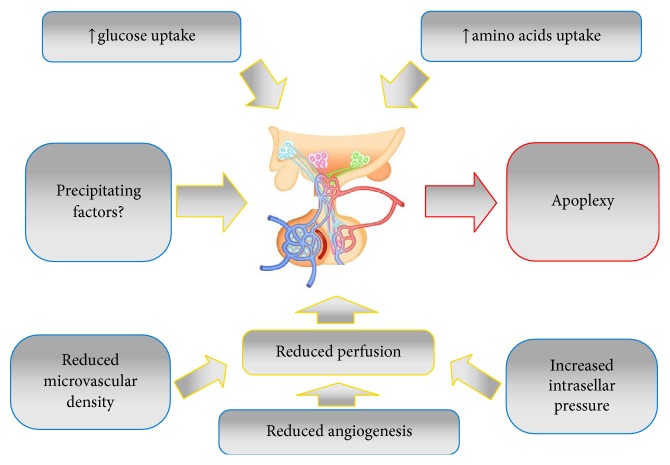
Pathophysiology of pituitary apoplexy.

The pathophysiology of pituitary apoplexy is not fully understood, but intrinsic features of pituitary tumour make it prone to bleed and undergo infarction (Figure 1). Indeed, pituitary adenomas have a high-energy requirement but a limited expression of angiogenic factors and a reduced vascular density and, therefore, a limited blood supply (Figure 1) [13]. As a consequence, any event that alters the balance between tumour perfusion and tumour metabolism may cause an acute ischemia or infarction (Figure 1). Moreover, an increased intratumoural and intrasellar pressure could concur to the reduction of tumour perfusion, further contributing to ischemia's pathomechanisms. Constitutional vascular fragility could also make pituitary tumours more susceptible to haemorrhage [14, 15].

In terms of triggering factors, the use of anticoagulants is reported in more than a quarter of patients [16–18]. New oral anticoagulants may also be involved [19, 20]. A precipitating role has been attributed also to the use of dopamine agonists (either at the beginning or after discontinuation of therapy) [21–28] and to oestrogen administration [29]. Angiographic and surgical procedures, especially cerebral angiography and cardiac or orthopaedic surgery, have been reported to be associated with pituitary apoplexy [30–35]. In other cases, it has been diagnosed within hours or days after traumatic brain injury [36] or pituitary radiosurgery [37]. Pituitary apoplexy has been also described in pituitary tumour patients undergoing hormone stimulation testing (with insulin, TRH, GnRH or GHRH and, more rarely, CRH), probably because of an imbalance between the test-induced metabolic demand and the blood supply modulation within the pituitary tumour [38–49].

Unlike previous studies, a recent publication suggests that diabetes and arterial hypertension do not predispose patients to pituitary apoplexy [16].

Finally, germline AIP gene mutations have been associated with apoplexy predisposition, probably because of the rapid growth of the pituitary tumour [50].

## 3. Clinical Features

Pituitary apoplexy is more frequent in men and during the 5th and 6th decades of life [10, 51]. Moreover, it has been suggested that a large pituitary tumour size is associated with a significantly increased risk of apoplexy [16, 52].

Pituitary apoplexy is, by definition, symptomatic and its clinical presentation can be acute or slowly progressive (subacute), depending on bleeding extent, oedema extension, and necrotic evolution [53]. Patients with symptomatic apoplexy complain of sudden and intense headache, rapid and dramatic visual impairment, vomiting, and, in the most severe cases, altered state of consciousness [54]. Headache is mainly retroorbital or frontal, violent and lancinating, and resistant to analgesics and is generally unlike other headaches patients commonly experience [55]. On the other hand, subclinical intratumoural haemorrhage can be incidentally detected by routine neuroimaging in about 25% of patients with pituitary adenomas [6, 56–58]. This circumstance does not represent a clinical emergency and management will depend on tumour's hormonal activity and size.

The diagnosis of pituitary apoplexy can be challenging, especially if the underlying pituitary tumour is still undiagnosed, because the above-mentioned symptoms occur also in patients with subarachnoid haemorrhage (SAH), bacterial meningitis, or cerebral ischemia [59]. Therefore, the main issue in the management of pituitary apoplexy is the misdiagnosis at admission, which can result in an inappropriate therapeutic approach. Thus, a correct interpretation of patient's history and of brain imaging can contribute to making the right diagnosis.

At presentation, the majority of pituitary apoplexy patients (nearly 80% of them) show one or more anterior pituitary hormone deficiencies [51, 54, 55]. Central hypoadrenalism has been reported in more than 70% of the patients and can have acute critical clinical consequences [4, 51, 54]. Since headache, visual impairment, hypotension, nausea, and, sometimes, hemodynamic shock may require rapid administration of high doses of glucocorticoids, endocrine investigations, for example, the biochemical evaluation of adrenal function, should be carried out preliminarily. Hyponatremia has been reported in up to 40% of patients, although it has been shown to be less common in some series [4, 51, 59–61]. Hyponatremia can be due to either severe hypocortisolism or inappropriate antidiuretic hormone secretion caused by hypothalamus irritation [62].

Thyrotropin (TSH) and gonadotropins (LH and FSH) deficiencies are observed in around 50% and 75% of patients, respectively, but they generally become clinically relevant only after several months when directly caused by pituitary necrosis [4, 51, 54].

The pathogenesis of the apoplexy-related hormone deficiencies is relatively complex and often multifactorial. In some cases, the pituitary function was already compromised before the apoplectic event because of the presence of a pituitary tumour [51, 54]. In many other cases, the endocrine impairment is directly due to the sudden and massive necrosis of pituitary tissue. Indeed, pituitary apoplexy causes a dramatic increase in intrasellar pressure, compressing the portal circulation, the pituitary stalk, and the pituitary gland, resulting in further damage of normal residual tissue [9, 63].

As suggested by the study of Zayour et al., serum PRL levels could be an excellent marker, with inverse relationship, of apoplexy extension and a reliable prognostic predictor of pituitary functional recovery. Accordingly, patients with low serum PRL levels at presentation would have the highest intrasellar pressure and they would be the least likely to recover from existing hormonal deficiencies after decompressive surgery [63]. It is worth mentioning that pituitary apoplexy can occur also in hormonally active pituitary adenomas. In these cases, it is possible to have a transient or persistent clinical and biochemical resolution of signs and symptoms of hormone hypersecretion after pituitary apoplexy [55, 64–67].

Visual impairment and ocular motility abnormalities are frequent [6, 51, 53, 54, 68]. Variable degree of visual impairment may be observed in more than 80% of the patients, with hemianopsia reported as the most common finding. Visual acuity loss, even to complete blindness, can rarely occur within a few hours from headache onset. About half of the patients report diplopia, and some of them have ocular paresis due to functional impairment of the third, sixth, or, less frequently, fourth cranial nerve [11, 51, 54, 69, 70]. The third cranial nerve is the most frequently affected, resulting in ptosis, mydriasis, and limited eye movements in adduction [54, 71, 72]. Visual field evaluation, especially its on-going evolution, ideally performed by an ophthalmologist or neuroophthalmologist, is critical not only for the diagnosis but also for planning the therapeutic approach modalities and, to some extent, for predicting prognosis. Visual function evaluation should include the assessment of (i) visual acuity, (ii) visual field defects, preferably detected by computed perimetry or by dynamic nonautomated Goldmann field analysis, and (iii) ocular motility. According to UK guidelines [59], severely impaired visual acuity and severe and persistent visual field defects are generally an indication for surgery, whereas isolated ocular paresis is not. Patients with mild visual deficits at presentation should be monitored because they could rapidly evolve.

## 4. Radiological Findings

**Figure 2:**
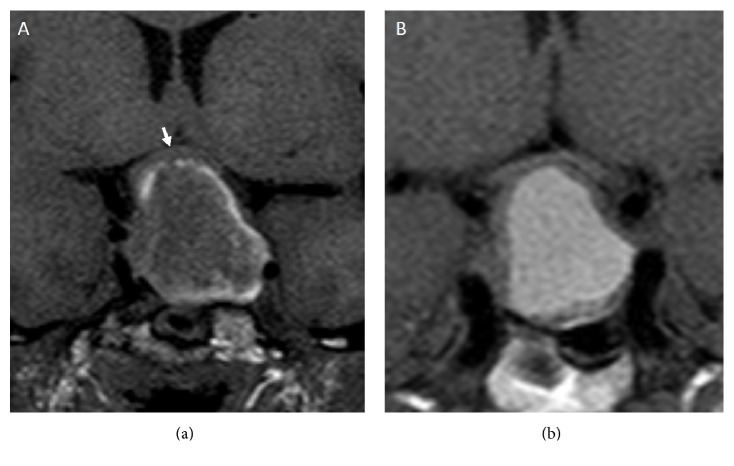
Coronal T1-weighted MRI images in two patients with pituitary apoplexy showing (a) intrasellar and suprasellar mass with a peripheral high signal intensity ring due to methemoglobin and slightly compressing the optic chiasm (white arrow) and (b) intrasellar and suprasellar mass with diffuse central high signal intensity.

**Figure 3:**
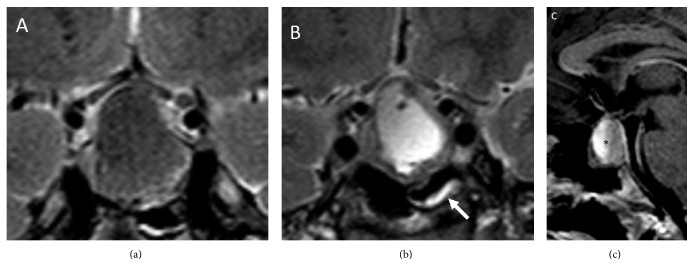
Coronal T2-weighted MRI images ((a) and (b)) and sagittal T1-weighted image (c) in patients with pituitary apoplexy showing (a) the low-signal haemorrhagic content of the pituitary mass, (b) the high-signal cystic area inside the lesion and the focal mucosal thickening of the sphenoid sinus (white arrow), and (c) a fluid-fluid intralesional level (asterisk) which is pathognomonic of pituitary apoplexy.

**Figure 4:**
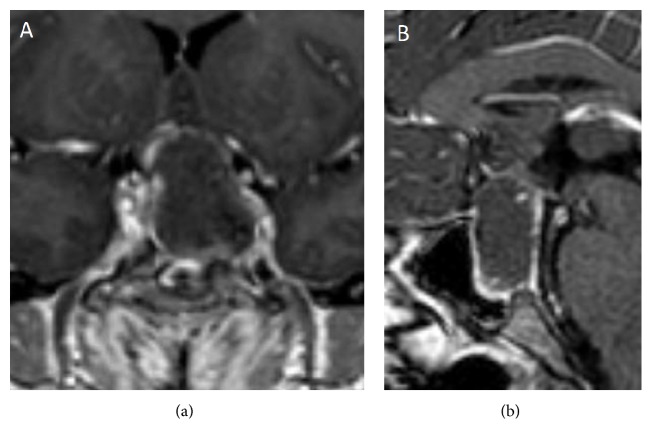
Coronal (a) and sagittal (b) T1-weighted MRI images after gadolinium administration show a moderate “ring” enhancement in a patient with pituitary apoplexy.

Haemorrhage and/or necrosis within a pituitary tumour are frequently incidentally observed by Magnetic Resonance Imaging (MRI) or Computed Tomography (CT). They are often asymptomatic, configuring the subclinical pituitary apoplexy, and occur in 14–22% of patients with a pituitary macroadenoma. On the contrary, massive haemorrhage is diagnosed in only 0.6–9.0% of cases and usually induces remarkable clinical consequences [73–75]. In patients with pituitary apoplexy, both CT and MRI show typical heterogeneous intrasellar and/or suprasellar lesions, with the coexistence of solid and haemorrhagic areas [76, 77]. CT is able to demonstrate alterations of pituitary parenchyma already during the acute phase of apoplexy, showing hyperdense intralesional areas due to recent bleeding. Later, during the subacute or chronic phase, hypodense areas can be visible due to progressive degradation of heme products, resembling other lesions with necrotic or cystic components. CT is also able to detect SAH or cerebral ischemia, which represent the most severe intracranial complications of pituitary apoplexy and have a critical role in the short-term management [76]. Nevertheless, first-line imaging technique for pituitary apoplexy diagnosis is MRI. After the first 12–48 hours from clinical presentation, MRI is more sensitive than CT for intralesional bleeding detection [76]. Precontrast T1-weighed scan can demonstrate intralesional areas of high intensity signal. This finding is due to the presence of methemoglobin resulting from subacute bleeding and it is generally arranged in the periphery of the lesion (Figure 2). On T2-weighted scans, the haemorrhagic areas frequently show low signal, whereas cystic areas are characterized by high signal intensity (Figures 3(a) and 3(b)). In both sequences, it is possible to find a fluid-fluid intralesional level (Figure 3(c)), in which the lower area corresponds to the red blood cells sediment, while the cranial one is constituted by free extracellular methemoglobin. After contrast injection, a peripheral “ring” enhancement is generally observed (Figure 4). MRI also shows cavernous sinus infiltration and optic chiasm compression [76]. Special techniques, such as T2^*∗*^-weighted Gradient Echo (GE) and Diffusion-Weighted Images (DWI), can usefully complete conventional MRI imaging [78]. T2^*∗*^-weighted GE MRI images are particularly useful in the identification of haemorrhagic focus in a pituitary macroadenoma, both in the acute phase and in the chronic phase. In these images, heme degradation products are markedly hypointense. In rare cases, these sequences show a leptomeningeal hemosiderosis, a real hemosiderinic tattoo, due to repeated bleeding of a pituitary tumour. On the other hand, DWI images allow early detection of necrotic areas, markedly hyperintense, in the tumour. In pituitary apoplexy patients, some authors reported the thickening of the sphenoid sinus mucosa (Figure 3(b)) [79]. It can occur during the acute phase of pituitary apoplexy and generally improves spontaneously. This thickening does not indicate infectious sinusitis and is not a contraindication to the surgical transsphenoidal route [80]. However, although neuroradiological evidence of pituitary bleeding strongly suggests a pituitary apoplexy, this is not pathognomonic. Indeed, other sellar lesions such as metastasis, craniopharyngioma, or hypophysitis can be associated with bleeding and, therefore, neuroradiological findings must always be correlated to clinical, anamnestic, and laboratory data.

A correct neuroradiological diagnosis can be difficult in patients with pituitary apoplexy and SAH. SAH can be caused by the apoplexy of the gland, but, considering the known association between pituitary adenoma and intracranial aneurysm [81], it is mandatory to exclude the presence of an intracranial aneurysm by a CT angiography or Digital Subtraction Angiography (DSA) [82].

The rare association between a ruptured aneurysm with SAH and pituitary apoplexy [83] as well as the rupture of an aneurysm embedded within a pituitary adenoma with a clinical evidence of epistaxis has also been reported [84]. Suzuki et al. reported a case of pituitary apoplexy caused by an unsuspected aneurysm bleeding into a pituitary adenoma and in which catastrophic intraoperative haemorrhage occurred [85]. Chuang et al. described a case of a coincidental pituitary adenoma and a giant ruptured intracavernous internal carotid artery (ICA) aneurysm [86]. On the other hand, ICA aneurysms can extend into the sella, simulating pituitary adenomas. The differential diagnosis, also in these cases, requires a preoperative CT angiography or DSA and the identification of associated radiological signs such as the erosion of the adjacent bony wall around the cavernous sinus or the presence of lamellar/circumferential calcification within the wall of the aneurysm. Nevertheless, the thrombosis of an intracavernous ICA aneurysm is reported as a potential catastrophic trap for the surgeon [87]. According to literature data, a critical evaluation of neuroradiological features is crucial in order to avoid catastrophic surgical decompression. Therefore, the possibility of a vascular lesion simulating or associated with a pituitary adenoma should be always taken into consideration by surgeons approaching a sellar mass.

## 5. Therapeutic Strategies

The best approach to the patient with pituitary apoplexy is extremely controversial. Indeed, early surgery is considered necessary in patients with consciousness state deterioration or in case of severe visual loss for optic chiasm compression. Nevertheless, increasing evidences show that a more conservative management can ensure favourable neuroophthalmological and endocrinological outcomes, at least in patients with moderate or spontaneous remission of visual impairment [56, 87–91]. In these cases, hydrocortisone administration (100–200 mg in bolus) is indicated also when signs or symptoms of hypoadrenalism are absent. Hydrocortisone infusion should be continued for 48 hours, decreasing the doses gradually (2–4 mg/hour in continuous infusion i.v. or 50–100 mg every 6 hours i.m.), according to clinical course [59]. If nausea and/or vomiting are absent or disappear, steroid therapy can be also challenged orally after few days (hydrocortisone 10 mg, 1 tablet tid, or 25 mg cortisone acetate, 1 tablet bid). Dexamethasone administration (4 mg i.m., every 12 hours), associated with lower-dose hydrocortisone infusion, has been also proposed in presence of visual loss or ophthalmoplegia [59]. However, if the patient is haemodynamically stable, steroid treatment should start after sampling for routine chemistry and endocrine parameters determination, while in presence of hemodynamic instability the glucocorticoid therapy should be promptly started. If endocrine evaluation performed before treatment shows low FT4 levels, replacement with L-thyroxine is recommended. It is worth noting that L-thyroxine substitution can unmask subclinical hypoadrenalism and, for this reason, hydrocortisone treatment should be started previously or at the same time [92–94].

Regarding the surgical approach, some studies show that rapid decompression of optic pathway generally associates with favourable outcome in patients with visual impairment, especially if progressively worsening [95]. Surgical decompression normalizes visual acuity in about one-half of cases and improves it in 6–36% of cases [4, 96]. However, the impairment of the III, IV, or VI cranial nerve can be permanent and surgery could not induce a significant improvement of ocular motility. On the other hand, isolated ophthalmoplegia is not an indication for surgery [9]. External ventricular drainage should be performed if intracranial pressure increases leading to changes of consciousness state. If an urgent approach is not required, surgery may be postponed until hemodynamic stabilization has been reached [97]. In some cases, it can become unnecessary for the spontaneous shrinkage of the pituitary mass. Some authors suggested that an early surgical treatment, within 7-8 days from the onset of symptoms, is associated with a better neuroophthalmological and endocrinological outcome [11, 51, 89, 97, 98]

**Figure 5:**
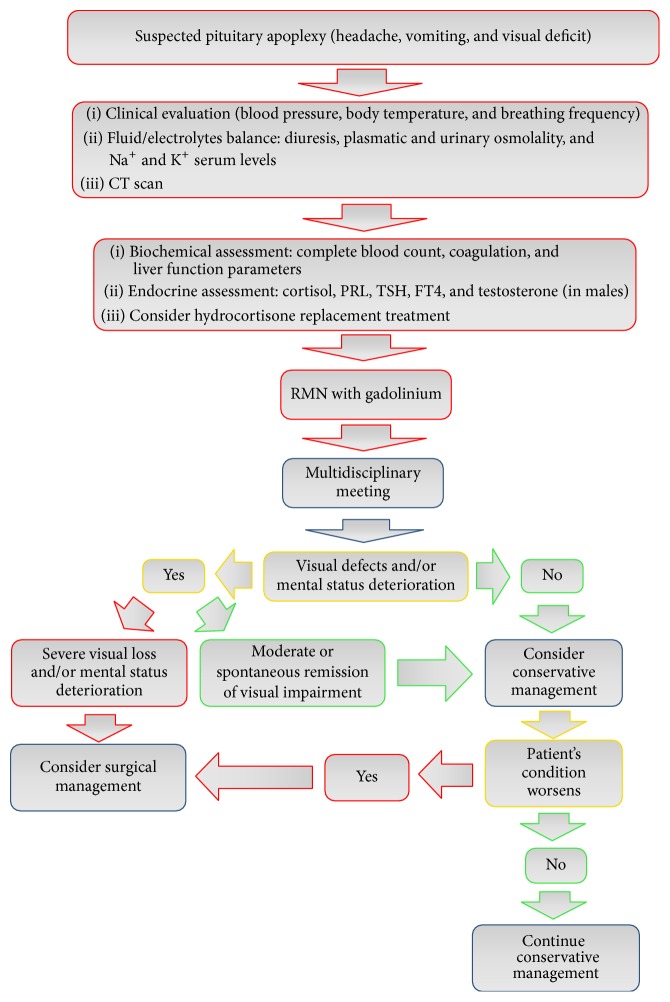
Algorithm for the management of pituitary apoplexy.

Recently, Rajasekaran et al. proposed a diagnostic and therapeutic algorithm aimed at identifying the best therapeutic approach [59]. Accordingly, we suggest that management of patients with pituitary apoplexy should be based on a multidisciplinary evaluation which involves endocrinologists, neuroradiologists, neurosurgeons, and neuroophthalmologists, referring the patient to surgery only if severe visual field defect or consciousness state impairment occurs (Figure 5). In addition, Rajasekaran et al. have also introduced a Pituitary Apoplexy Score (PAS), which should be calculated at patient's admission. It is based on three neuroophthalmic parameters (visual acuity: 0, 1, and 2; visual field defect: 0, 1, and 2; and ocular paresis: 0, 1, and 2) and on Glasgow Coma Scale (GCS: 0, 2, and 4). This scoring system ranges from 0 to 10, with a higher score indicating extensive neuroophthalmic impairment [59]. Bujawansa et al. retrospectively applied the PAS system to their patient cohort. In this study, PAS was calculated in 84% of 55 patients with pituitary apoplexy evaluated retrospectively. At admission, 87% of them complained of headache, 47% of diplopia, and 36% of visual field impairment. Surgical decompression was performed in 32 cases within 7 days after diagnosis, while a conservative strategy was adopted in the other patients. Overall, visual field and ocular paresis improved in 85% and in 91% of cases, respectively, but recovery of visual field and endocrine function was not significantly related to therapeutic strategy. Surgery was necessary in all patients with PAS ≥ 4 [99].

Finally, Jho et al. recently proposed the Pituitary Apoplexy Grading System: grade 1 patients are asymptomatic and pituitary apoplexy is detected incidentally (“subclinical” apoplexy); grade 2 patients present endocrine dysfunction; grade 3 patients complain of headache; and grade 4 patients show ocular paresis, while grade 5 subjects present acute visual deficits or altered mental status, precluding visual assessment. The authors suggested that endocrine dysfunction recovers more frequently in grades 1–3 patients, whereas early surgery is recommended in those with higher grading [100]. However, subclinical apoplexy could have been underestimated in this study since it was based on a retrospective surgical series.

## 6. Prognosis

Endocrine and neuroophthalmological prognosis of pituitary apoplexy inevitably depends on the appropriateness of the management during the acute and subacute phase of the disease. Indeed, patients with severe neurological or ophthalmological impairment can remarkably improve if correctly approached, while the outcome of those with mild sings/symptoms can be worse if the diagnosis is delayed or they are incongruously treated.

The middle- and long-term outcome of patients with pituitary apoplexy is strictly related to (i) the pathomechanisms of pituitary damage, (ii) the involvement of optic tract and oculomotor nerves, and (iii) the occurrence of SAH [101]. At the same time, in our opinion, a multidisciplinary management as well as a skilled and dedicated surgical team could affect the outcome of pituitary apoplexy. Some evidences suggest that an early surgical approach would increase the chances of visual impairment recovery [11, 53, 82, 102]. Turgut et al., by reviewing the literature of pituitary apoplexy patients presenting with monocular or binocular blindness, concluded that delayed surgery could have a negative impact on visual recovery [82]. On the contrary, the resolution of ocular paresis is not related to the surgical timing [11, 73, 92]. However, there are controversial data on the influence of different treatment modalities, conservative or surgical, on visual outcomes. Indeed, spontaneous visual improvement has also been described [64, 82, 90, 103].

SAH following pituitary apoplexy is due to the bleeding into the suprasellar cistern. It is a rare event, which, however, increases the risk of brain stroke due to secondary vasospasm [36, 104]. This complication is mainly responsible for neurological consequences and consciousness impairment.

Regarding endocrine deficiencies, signs and symptoms of hypocortisolism are generally observed in the early stage after apoplexy onset, while hypothyroidism, hypogonadism, or growth hormone deficiency is extremely frequent and may occur progressively during weeks, months, or years. Accordingly, a periodic reevaluation of anterior pituitary function is recommended [37]. On the contrary, diabetes insipidus is extremely rare, being present in less than 5% of patients [105]. The impairment of the pituitary function can be due to the haemorrhagic destruction of the gland or due to the compression of residual normal tissue. In the former condition, hypopituitarism is frequently permanent, while in the latter one recovery of endocrine function can be obtained by debulking of the pituitary mass [63, 106]. However, in terms of endocrine outcome, some studies showed that pituitary hormones deficiencies consequent to pituitary apoplexy infrequently recover and the persistence of hypopituitarism seems to be not related to management modalities [4, 96, 99, 101, 107].

Finally, Pal et al. reported that residual pituitary tumour regrowth occurs in 21.4% of patients within 5 years after apoplexy; therefore a periodic radiological evaluation of pituitary gland is recommended [108]. This risk appears to be comparable in secreting and nonsecreting tumours and is lower in patients treated with radiotherapy [108]. Recurrent pituitary apoplexy is a rare event, although few cases have been reported [109]. It appears to be more common in patients managed conservatively than in those surgically treated [52].

## 7. Conclusions

Pituitary apoplexy is a diagnostic and therapeutic challenge, even because largely adopted specific guidelines are lacking. In many cases, it is a serious medical emergency that requires a multidisciplinary approach, which involves endocrinologists, neuroradiologists, neurosurgeons, and neuroophthalmologists. The outcome is difficult to predict and highly variable [51, 110, 111]. The optimal time of surgery and its impact on visual impairment are still matter of debate. Surgical decompression may be necessary when the visual deficits are rapidly progressive and high-dose corticosteroid therapy or CSF shunt placement is ineffective. Surgery is indicated within 7-8 days after diagnosis when the visual field defects do not improve or worsen or even later if ophthalmoplegia persists. Pharmacological treatment with high-dose steroids is the approach of choice when symptoms are mild. Finally, long-term hormone replacement treatment can be necessary if hypopituitarism occurs, but pituitary function should be reassessed over the medium term and long term, because resolution of existing deficiencies or development of new ones has been reported [112].
